# Patient with Down syndrome and relapsed acute lymphoblastic leukemia with sustained remission despite only partial R3 chemotherapy

**DOI:** 10.1002/ccr3.3678

**Published:** 2021-02-12

**Authors:** Zhongbo Hu, Kristen A. VanHeyst, Jignesh Dalal, Lisa Hackney

**Affiliations:** ^1^ Department of Pediatrics Division of Pediatric Hematology Oncology Rainbow Babies and Children’s Hospital at University Hospitals Cleveland Medical Center Cleveland OH USA

**Keywords:** ALL R3, Down syndrome, Relapsed acute lymphoblastic leukemia, remission

## Abstract

DS‐ALL has a higher rate of relapse and treatment‐related mortality. The newer immunotherapies are potentially better options. Relapsed ALL with positive MRD has a poor prognosis. Transient long‐term remission after ALL relapse due to partial chemotherapy combined severe infection is rare.

## Graphical abstract

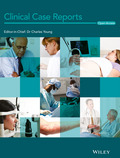

## 1 INTRODUCTION

DS‐ALL patients have a higher rate of relapse and treatment‐related mortality. We describe a DS‐ALL patient with late bone marrow relapse who was treated per British ALL R3 reinduction chemotherapy. The patient remained in remission for approximately two years after therapy was discontinued due to severe systemic infections.

The current treatment regimens for acute lymphoblastic leukemia (ALL) have successfully attained cure rates of approximately 90%‐95% in children, but still with poor overall long‐term survival in adults of about 40%.^1^, ^2^, ^3^ It still remains a significant challenge to improve the survival of patients with relapsed ALL. Patients with relapsed ALL have a suboptimal prognosis.^1^, ^4^ Down syndrome patients (DS) with relapsed ALL have even worse outcomes. DS itself was proven to be an independent prognostic factor of outcome after ALL relapse.^5^ ALL patients with DS have a higher rate of relapse and treatment‐related mortality, with an 8‐year cumulative incidence of relapse of 26% comparing with 15% in non‐DS patients and a 2‐year treatment‐related mortality of 7% with 2% in the latter.^6^ There are several case reports of patients with leukemia who attained spontaneous remission after severe infection.^7^, ^8^, ^9^ There are no reports of a patient with relapsed leukemia with sustained remission following cessation of therapy secondary to severe infections, except an older patient with relapsed FLT3 internal tandem duplication mutant acute myeloid leukemia whose disease underwent spontaneous remission for 35 days without precipitating cause.^10^

We describe a case of a 37‐year‐old man with Down syndrome and ALL (DS‐ALL) who had a late bone marrow relapse and sustained remission for 2 years after only receiving two cycles of ALL R3 salvage chemotherapy due to severe systemic infections.

## 2 CASE PRESENTATION

A 30‐year‐old male with Down syndrome presented with fever, cough, and shortness of breath in November 2013. He was managed in our pediatric unit because of current favorable outcomes for Adolescents and Young Adults (AYA) with ALL treating with pediatric Children's Oncology Group (COG) protocols.^11^ His complete blood count (CBC) showed pancytopenia with a white blood cell (WBC) count of 1.4 x 10^9/L, hemoglobin of 8.3 g/dL, and platelet count of 32 x 10^9/L with occasional circulating blasts. Bone marrow aspirate and biopsy confirmed the diagnosis of acute lymphoblastic leukemia with 73% blasts which co‐expressed CD34, CD19, CD10, and terminal deoxynucleotidyl transferase (TdT) with aberrant expression of dim CD33 by flow cytometry, which was consistent with B‐precursor ALL. Cytogenetic analysis was not successful but fluorescence in situ hydridization (FISH) from a bone marrow sample obtained on day 17 during Induction chemotherapy as per the COG study AALL1131 showed nuc ish (ETV6x2,RUNX1x3)[250] which was a normal signal pattern for constitutional trisomy 21 with two ETS Variant Transcription Factor 6 (ETV6) signals and three Runt‐related transcription factor 1 (RUNX1) signals. Diagnostic cerebrospinal fluid was negative for blasts.

The patient was treated per COG high‐risk protocol AALL1131, Down syndrome arm due to National Cancer institute (NCI) risk factor for age above 10 years old. He had a rapid early response with a M1 marrow at Day 17, and he achieved complete remission with complete haematological recovery for about six months after induction with negative minimal residual disease (MRD). Although, during induction phase he had a complicated treatment course including a few episodes of sepsis, one of which included multi‐organ failure for which he was in the Medical Intensive Care Unit (ICU), and peripherally inserted central catheter (PICC) line associated deep vein thrombosis (DVT). Following the end of his treatment, his platelet count never normalized and ranged between 90x10^9/L and 112x10^9/L. At his lowest level of thrombocytopenia to 48x10^9/L in September 2017, which was four years after the initial diagnosis, a repeat bone marrow aspirate and biopsy were obtained and showed a 90% cellular bone marrow with 82% blasts, consistent with late bone marrow relapse. FISH showed cytokine receptor like factor 2 (CRLF2) translocation positive in 63.5% of cells and was positive for deletion of CDKN2A on chromosome 9p beside his constitutional trisomy of chromosome 21. Central nervous system (CNS) was negative. This relapse was treated with Reinduction chemotherapy per ALL R3 protocol (Dexamethasone 20mg/m2/day day 1‐5 & 15‐19, Mitoxantrone 10mg/m2/dose day 1‐2, Vincristine 1.5mg/m2/dose from day 3, then weekly; PEG‐asparaginase 2500U/M2 day 3 and 18; Intrathecal Methotrexate day 1 and 8). Post‐reinduction day 29 MRD was 0.2% (Table 1).

**Table 1:** Patient's bone marrow aspiration time points and characteristics

Date	BM time point	blast %/ MRD	Immunophenotype	FISH	Cytogenetics
11/21/2013	Initial diagnosis	73%	CD34, 19, 10, TdT(+); CD33 dim		Not sent
12/2/2013	Day 10 of induction	9%			failed culture
12/9/2013	Day 17 of induction	/0.1%		nuc ish (ETV6x2,RUNX1x3)[250]	
12/24/2013	Day 31 of induction	/0.008%			failed culture
3/25/2016	Finished chemotherapy				
9/19/2017	Found relapse	82%	CD34,19, 10, 304 (+)	>Positive for CRLF2 translocation (63.5% of cells) (Univ of Cincinnati) >Positive for deletion of CDKN2A on chromosome 9p. >Positive for trisomy of chromosome 21 (constitutional).	47,XY, +21c[25]
10/23/2017	Day 29 post‐reinduction	/0.2%			
11/6/2017	Day 43 post‐reinduction	/0.03%			
1/2/2018	post‐consolidation of ALL R3	0%	/	FISH negative for 9p deletion; nuc ish (CDKN2A,CEP9)x2 [250]	47,XY, +21c[25]
3/15/2018	25 weeks after relapse	0%	/	not performed	47,XY, +21c[25]
8/30/2018	50 weeks after relapse	0%	/	negative for 9p deletion; nuc ish (CDKN2A,CEP9)x2 [250]	47,XY, +21c[25]
2/3/2020	2 years and 20 weeks after first relapse	90%	CD34, 19, 20, 10; 50% (+) for CD22; CD304 Dim‐moderate		

He continued to receive Consolidation chemotherapy per ALL R3 protocol (Intrathecal Methotrexate day 1, Dexamethasone 6mg/m2/day day 1‐5, methotrexate 1000 mg/m2/dose day 8 with Leucovorin rescue, Vincristine 1.5 ng/m^2^/dose day 3, PEG‐asparaginase 2500 U/m^2^/dose day 9, Cyclophosphamide 440mg/m2/dose day 15‐19, Etoposide 100mg/m2/dose day 15‐19), after which his bone marrow had negative MRD. While hospitalized for Intensification chemotherapy, he developed sepsis, necrotizing pneumonia, and severe bilateral pleural effusions. Bronchoalveolar lavage (BAL) revealed Candida albicans. Computerized tomography (CT) imaging also revealed near complete splenic infarct, right renal perfusion deficits with signs of liquefaction and a segmental area of abnormal small bowel wall enhancement and concern for focal small bowel wall defect. He also developed delirium related to his severe illness and prolonged hospitalization. Due to severe illness, his chemotherapy was held during a three‐month period. A bone marrow after three months continued to demonstrate negative MRD. Once he became more clinically stable, chemotherapy was attempted with intermediate dosed Methotrexate but this hospital course was complicated by infection necessitating removal of his broviac, fluid overload, and a persistent cavitary lung lesion. At this point, a decision was made by his family and treatment team to stop chemotherapy. About 50 weeks after his diagnosis of relapsed disease, a repeat bone marrow continued to demonstrate negative MRD. He was followed closely as an outpatient. Unfortunately, he presented with acute onset psychosis and thrombocytopenia about 2 years after stopping his treatment and bone marrow was consistent with relapsed disease. Family opted for no further therapy, and unfortunately, the patient passed away 7 weeks later.

## 3 DISCUSSION

Despite current long‐term remission with leukemia‐free survival of ALL reaching above 90% in children,^1^, ^12^ relapsed ALL and adult cases are still the most important obstacles for the cure of ALL patients. The relapse rate is about 10%‐15% of ALL in children, but substantially higher (about 25%‐30%) in high‐risk subgroups.^13^, ^14^ Almost 50% of adult ALL patients experience relapse.^15^ DS patients with ALL even have high relapse rate,^6^ partially due to poor tolerance to anti‐leukemia drugs.^16^ DS patients more frequently develop severe infectious complications.^16^

Although high‐dose Mitoxantrone plus Cytarabine was effective in achieving remission in refractory leukemia, the duration of the remission was only about half a year^17^ and still about 50% of ALL patients do not respond to salvage therapy prior to the ALL R3 protocol.^18^ The British ALL R3 protocol utilizes Mitoxantrone to confer a significant response in progression‐free and overall survival in children with relapsed ALL.^4^ Our patient, who had a bone marrow relapse about 20 months after the completion of his initial chemotherapy, was considered to have late bone marrow relapse (defined as relapse occurring 36 months after the first diagnosis or more than 6 months after the end of front‐line therapy). He was treated as per the ALL R3 protocol and had positive MRD of 0.2% after Reinduction and negative MRD after Consolidation. Per the ALL R3 protocol, patients with B‐precursor ALL with late bone marrow relapse and low MRD (<0.01%) at the end of induction had favorable outcomes with chemotherapy without undergoing stem‐cell transplantation. Patients with higher MRD (>0.01%) required allogeneic stem‐cell transplantation. But after long‐term follow‐up of the late bone marrow relapse patients for 7 years, the second relapse rate was about 23% in the high MRD group even after stem‐cell transplant. We had discussed this as a group together with patient's parents. Both parents and all physicians thought that our patient's transplant‐related mortality (TRM) would be too high given his toxicities with initial chemotherapy and his Down syndrome status. We chose to continue chemotherapy per ALL R3. In the low MRD group with chemotherapy, second relapse rate was about 27%.^19^ The progression‐free survival at 5 years was significantly lower in the high MRD group comparing with that in the low MRD with 56% versus 72%.^19^ Our case is unique in that the patient was still complicated with several systemic infection after only receiving two phases of chemotherapy per the ALL R3 protocol for his first relapse, but remained in remission for nearly two years.

Patients with Down syndrome are well‐known to have 10‐ to 20‐fold increased risk of developing B‐cell precursor ALL.^20^ The Ponte di Legno study group showed that DS‐ALL patients had higher 8‐year cumulative incidence of relapse than non‐DS patients (26% vs 15%).^6^ AYA patients with ALL have favorable outcomes when they are treated with pediatric protocols.^11^ Treatment‐related mortality, primarily from infection, increased in all protocols in DS‐ALL, which made both the five‐year event‐free and overall survival inferior in patients with DS‐ALL.^21^, ^22^ Current COG protocols have made modifications to limit toxicities. Our patient had multiple infections during his initial chemotherapy per AALL1131. He also developed multiple severe infections after two phases of chemotherapy per the ALL R3 protocol following disease relapse. Given the significant toxicity that he experienced, a decision was made for cessation of therapy, which we thought would benefit the patient better and was approved by patient's parents. Over time, more options are becoming available for patients with relapsed ALL that confer less toxicity. Currently, antibody‐targeted therapies, such as bispecific (CD3/CD19) T‐cell‐engaging antibody Blinatumomab, Inotzumab, and CD19‐directed chimeric antigen receptor (CAR) T‐cell therapies, are major breakthroughs in the management of relapsed leukemia.^23^

Spontaneous or transient remission of acute lymphoblastic leukemia during severe infection has been described in a few cases and case series reports with averaged ten weeks in duration.^7^, ^8^, ^9^, ^24^ The proposed mechanism was due to increased cortisol production during stress or infection combined with an immune‐mediated process thereby generating anti‐leukemic effects.^7^, ^8^, ^25^ Cytokines, including tumor necrosis factor‐alpha, interferon‐alpha, and interleukin‐2, released during infection can directly inhibit the residual blast proliferation or through increased activity of T lymphocytes, macrophages, and natural killer cells leading to an anti‐leukemia effect.^26^, ^27^ Similar phenomena and mechanisms have been discussed and reported in several cases and case series in acute myeloid leukemia patients.^28^, ^29^ Our patient's long‐term remission may be attributed to a combination of the two phases of chemotherapy per ALL R3 protocol with the systemic stress related to his multiple severe infections.

## CONFLICT OF INTEREST

The authors declare no conflicts of interest.

## AUTHORS’ CONTRIBUTIONS

ZH: gathered the patient data, performed a literature review, and wrote the manuscript. KAV and LH: reviewed, corrected patient data and revised the manuscript. JD: was involved in overall supervision of the paper. All authors: read and approved the final manuscript.

## Data Availability

The data that support the findings of this study are available on request from the corresponding author. The data are not publicly available due to privacy or ethical restrictions.
